# IgG Glyco-Engineering to Improve IVIg Potency

**DOI:** 10.3389/fimmu.2018.02442

**Published:** 2018-10-23

**Authors:** Christine W. Bruggeman, Gillian Dekkers, Remco Visser, Naneth W. M. Goes, Timo K. van den Berg, Theo Rispens, Gestur Vidarsson, Taco W. Kuijpers

**Affiliations:** ^1^Sanquin Research and Landsteiner Laboratory, Department of Blood Cell Research, Academic Medical Center, University of Amsterdam, Amsterdam, Netherlands; ^2^Sanquin Research and Landsteiner Laboratory, Department of Experimental Immunohematology, Academic Medical Center, University of Amsterdam, Amsterdam, Netherlands; ^3^Sanquin Research and Landsteiner Laboratory, Department of Immunopathology, Academic Medical Center, University of Amsterdam, Amsterdam, Netherlands; ^4^Emma Children's Hospital, Academic Medical Center, University of Amsterdam, Amsterdam, Netherlands

**Keywords:** IgG, Fc glycan, glyco-engineering, FcγRs, IVIg

## Abstract

Intravenous immunoglobulins (IVIg) are used in the treatment of different autoimmune and inflammatory diseases, such as immune thrombocytopenia and hemolytic anemia. One of the modes of action of IVIg is preventing phagocytosis of autoantibody-opsonized blood cells by saturation of the Fc-gamma receptors of macrophages in spleen and liver. IgG contains a fixed glycan, which is in most cases biantennary, at the asparagine residue at position 297 in the Fc tail. This glycan consists of a core structure of N-acetyl glucosamine (GlcNAc) and mannose groups, variably extended with core fucose, bisecting GlcNAc as well as terminal galactose and sialic acid. This structural glycan influences the binding affinity of IgG to Fc-gamma receptors. By glyco-engineering, we generated monoclonal IgG antibodies with different Fc-tail glycans and tested both their opsonizing and blocking capacity in a phagocytosis assay of IgG-opsonized erythrocytes with human monocyte-derived macrophages. In contrast to a lack of effect in opsono-phagocytosis, these IgG glycovariants differentially inhibited the uptake of opsonized erythrocytes. The level of bisecting GlcNAc and galactosylation had unexpectedly larger impact than core fucosylation, and suggest that targeted modifications different from the core fucose may well improve the immunomodulating efficacy of IVIg treatment.

## Introduction

Intravenous immunoglobulin (IVIg) is pooled immunoglobulin G (IgG) from the plasma of thousands of donors. Besides the use as replacement therapy in patients with primary immunodeficiencies, it is used as immunomodulating agent for various autoimmune and inflammatory disorders, such as immune thrombocytopenia (ITP) and autoimmune hemolytic anemia (1–3).

The working mechanisms of IVIg as immunomodulating agent are not entirely understood. There are different working mechanisms hypothesized, such as blockade of the neonatal Fc receptor, direct interaction with the inhibitory Fc-gamma receptor (FcγR) IIb or saturation of the activating FcγRs. Since IVIg treatment is used for various diseases, it is possible that the mode of action differs per clinical setting (1–5).

In the 1980s, Imbach et al. showed that IVIg treatment of ITP patients resulted in a dramatic increase in platelet counts (6). When treating ITP patients with only the Fc part of IVIg, similar results were obtained, (7) indicating that the working mechanism is Fc-dependent (2, 7). To date, saturation of activating FcγRs expressed on macrophages and thereby inhibiting the uptake of autoantibody-opsonized platelets is one of the most prominent explanations for the beneficial effect of IVIg treatment in ITP patients (1–3).

All IgG subclasses contain a glycan at a conserved asparagine in the Fc tail. This glycan consists of a core structure of N-acetyl glucosamine (GlcNAc) and mannose, and can be variably extended with core fucose, bisecting GlcNAc, as well as terminal galactose and sialic acid (8). This variation affects the binding affinity of IgG to the different FcγRs (9–11), which may influence the working mechanism of IVIg (4, 12–15). The Fc glycan is of critical importance, as deglycosylated IVIg can no longer bind to FcγRs and thereby loses its activity in the treatment of diseases where FcγR binding is essential, such as ITP (2, 16).

The limited presence of a specific IgG fraction of the IVIg preparation may explain the fact that high concentrations of IVIg are required to obtain immunomodulatory effects *in vivo* (2, 12). In order to investigate the role of glycosylation on the inhibitory capacity of IgG, we produced differently glycosylated IgG antibodies to test their blocking capacity in the phagocytosis of antibody-opsonized erythrocytes by human macrophages.

## Methods

### Human samples

Peripheral blood from healthy volunteers was obtained in heparinized tubes. Written informed consent was obtained from all participants. The study was approved by the Medical Ethics Committee of the Academic Medical Center and performed in accordance with the Declaration of Helsinki.

### Antibody production

A combined vector encoding anti-TNP or anti-RhD IgG1 heavy chain and kappa light chain was cloned into pEE14.4 vector. All IgGs were produced by transient transfection of HEK-freestyle cells (Thermo Scientific), as previously described by Kruijssen et al. (17). The Fc glycan was engineered as published previously (14, 18).

### Culture of monocyte-derived macrophages

Monocytes were isolated from peripheral blood mononuclear cells and cultured into monocyte-derived macrophages, as previously described (13, 19). In short, monocytes were isolated using a CD14 MACS isolation kit and cultured for 9 days in IMDM, supplemented with 10% fetal calf serum, glutamine and antibiotics, containing either 10 ng/mL granulocyte macrophage-colony stimulating factor (GM-CSF) or 50 ng/mL macrophage-colony stimulating factor (M-CSF).

### Phagocytosis assay

Phagocytosis assay was essentially performed as described previously (13, 19). RhesusD (RhD) positive erythrocytes were stained with CFSE (Life technologies) and opsonized with polyclonal anti-RhD (RheDQuin, Sanquin, Amsterdam), at an optimal dose of 1.56 IE/mL, (19) or 10 μg/mL monoclonal anti-RhD (produced as described above), for 30 min at 37°C, or left unopsonized. After washing excess antibody away, the cells were added to the monocyte-derived macrophages in a ratio of 10:1. The M-CSF macrophages were incubated with the opsonized erythrocytes for 20 min, the GM-CSF macrophages for 2 h.

In some experiments, antibodies directed against an irrelevant antigen, anti-2,4,6-trinitrophenol (anti-TNP), with different Fc-glycoforms were added in the concentrations 0.1; 0.3; 1.0; 3.0, and 10 μg/mL, prior to the addition of erythrocytes to the macrophages. After phagocytosis, the non-phagocytosed red blood cells were lysed and the percentage of positive macrophages was determined by flow cytometry [FACS CANTO II (BD)].

### Statistical analysis

Statistical analysis was performed using GraphPad 7.04. Statistical tests that were used were multiple *t*-tests corrected for multiple comparisons using Holm-Sidak and one-way ANOVA, corrected for multiple comparisons using Dunnett.

## Results and discussion

**Figure 1:**
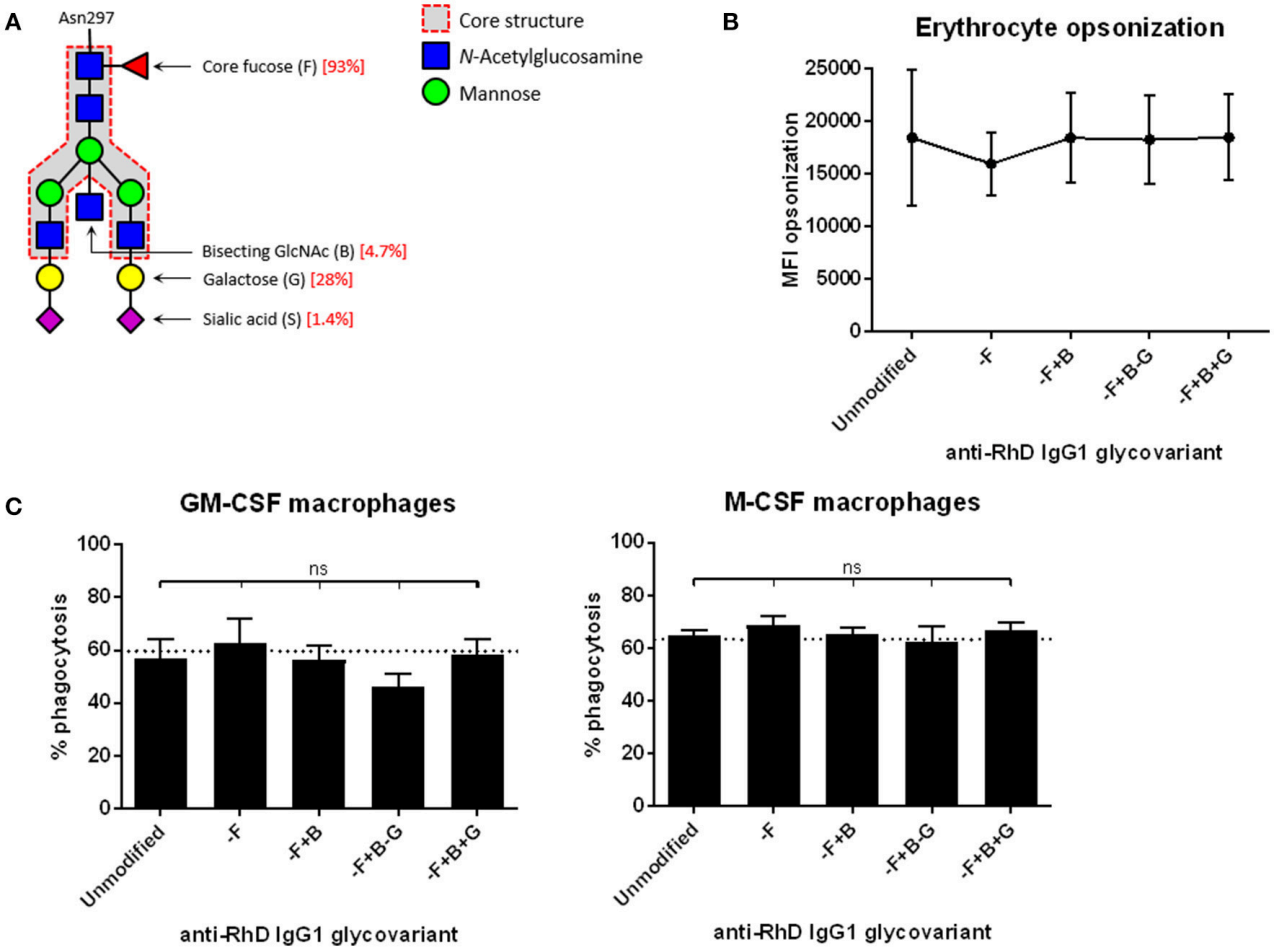
Monocyte-derived macrophages phagocytose erythrocytes that are opsonized with different anti-RhD IgG1 glycovariants to similar extent. **(A)** Composition of the bi-antennary glycan at position Asn297 in the IgG Fc domain. Percentages indicated in red are for the unmodified IgG1 variant (14). **(B)** Erythrocytes opsonized with monoclonal anti-RhD glycovariants (x-axis) were stained with goat-anti-human Ig to determine the amount of antibody deposition. Median fluorescence intensity (MFI) is shown on the y-axis. **(C)** Phagocytosis of anti-RhD opsonized erythrocytes by monocyte-derived macrophages cultured with GM-CSF (left) or M-CSF (right). Erythrocytes were opsonized with unmodified anti-RhD IgG1 or an anti-RhD IgG1 glycovariant (indicated on the x-axis). Percentage of positive macrophages is shown on the y-axis. **(B,C)** *n* = 6–10. Analysis is performed by one-way ANOVA, corrected for multiple comparisons using Dunnett.

To investigate the effects of antibody glycovariants on phagocytosis by macrophages, we engineered IgG antibodies with a variable glycan moiety and invariable, monoclonal antigen-specificity against RhesusD (anti-RhD). Typically, IgG unmodified for the glycan show 93% fucosylation, 5% bisecting GlcNac, 30% terminal galactose, and up to 2% sialic acid residues (Figure 1A), which is similar to normal human plasma-derived IgG (18).

We decreased the fucosylation (–F) to 27%, increased the bisecting GlcNAc (+B) to 53%, decreased or increased the extending galactose (– or +G), resulting in respectively 9 and 70% galactosylation, and increased sialic acid (+S) to 90%, compared to unmodified IgG1 (14, 18). These anti-RhD IgG1 Fc-glycovariants were used to opsonize RhD-positive erythrocytes. The level of antibody binding to the erythrocyte surface among these glycovariants was identical (Figure 1B). Erythrocytes opsonized with the various glycovariants were phagocytosed by monocyte-derived macrophages to the same extent as erythrocytes opsonized with unmodified IgG1 (Figure 1C). We had shown before that less opsonization results in less phagocytosis, irrespective of Fc-glycoform (13).

These findings contrast with our previous results with NK cell antibody-dependent cellular cytotoxicity (ADCC). With the same glyco-engineering tools, our previous studies had shown that IgG defucosylation had a major impact on NK cell function when used to opsonize erythrocytes as target cells (13). NK cells express FcγRIIIa as the only IgG receptor. Defucosylated IgG1 antibodies have a 10- to 40-fold increased affinity to FcγRIIIa (13, 14), which explained their enhanced effector function. However, cultured macrophages expressing a more complex spectrum of FcγRs did not show any altered effector function, i.e., opsono-phagocytosis, when defucosylated antibodies were used as opsonizing antibodies (13).

**Figure 2:**
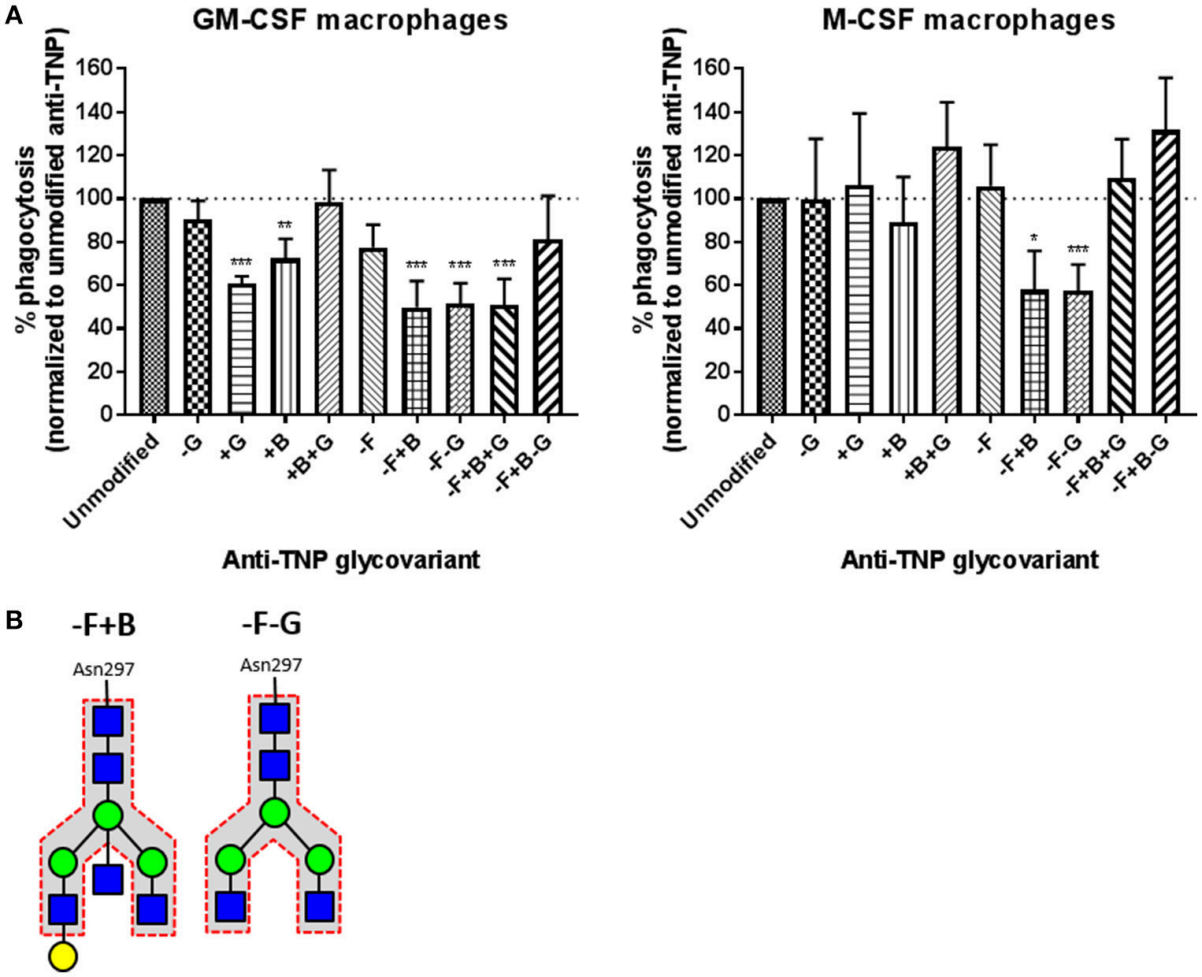
Anti-TNP IgG1 antibodies with low fucose low galactose (–F–G) and low fucose high bisection (–F+B) inhibit phagocytosis of anti-RhD-opsonized erythrocytes significantly better than unmodified IgG1 antibodies in both M-CSF and GM-CSF cultured macrophages. **(A)** Anti-TNP antibodies with different Fc-glycovariants (indicated on the x-axis) were used, at 1 μg/mL, to inhibit the phagocytosis of polyclonal anti-RhD opsonized erythrocytes by monocyte-derived macrophages cultured with GM-CSF (left) or M-CSF (right). Data were normalized to the percentage of macrophages that phagocytosed anti-RhD opsonized erythrocytes when inhibited with 1 μg/mL unmodified anti-TNP IgG1 (which resulted in about 30% phagocytosis compared to the unblocked control). Data represent mean and standard error of the mean (*n* = 3 batches, always tested in at least 4 times per batch). Glycovariants are compared to unmodified IgG, analysis is performed by multiple *t*-tests, corrected for multiple comparisons using Holm-Sidak, *p < 0.01; **p < 0.001; ***p < 0.0001. **(B)** Schematic representation of the bi-antennary Fc glycan of the two best blocking glycovariants.

In order to investigate the competition of soluble IgG on the IgG-mediated phagocytosis by human macrophages, we made 16 different IgG1 Fc-glycovariants with antigen-specificity against the irrelevant antigen TNP. Addition of these antibodies blocked the uptake of anti-RhD (polyclonal IgG purified from RhD-immunized individuals) opsonized erythrocytes by competing in a dose-dependent manner (Supplementary Figure 1), as expected from previous experiments (13, 19). We found two different glycoforms (–F+B, with 27% fucose and 53% bisecting GlcNAc but unchanged levels of galactose and sialic acid, and –F–G, with 27% fucose and 9% galactose, but unaltered levels of bisecting GlcNAc and sialic acid) to inhibit the phagocytosis of antibody-opsonized erythrocytes significantly stronger than unmodified IgG1 (Figure 1A), in both GM-CSF- and M-CSF-cultured macrophages (Figure 2). Although there was some donor-variation in phagocytosis capacity, three different glyco-engineered batches of monoclonal anti-TNP IgG1 consistently showed that the –F+B and –F–G glycovariants inhibited phagocytosis most. HPLC analysis showed that these antibodies contain a small fraction of dimers, but no multimers (Supplementary Figure 2A), which did not correlate with blocking capacity (Supplementary Figure 2B) (14). Although this suggests that dimer content did not affect the blocking capacity, we cannot completely rule out this possibility. The exact composition of the –F, –F+B, and –F–G glycoforms are shown in Supplementary Figure 3.

FcγRIIa is the predominant receptor to mediate the phagocytosis process in M-CSF-cultured macrophages, whereas in GM-CSF-cultured macrophages FcγRI and FcγRIIIa are mainly involved (19). Although there were no differences in binding affinities of the various glycovariants to FcγRI or FcγRII when measured by SPR (14), we here show that some glycovariants translated into blocking capacity in a cellular system with a more complex and dynamic spectrum of surface FcγR expression. Previous studies have shown that Fc glyco-engineering does not change the binding to the neonatal Fc receptor (FcRn) (20, 21).

Next to changes in binding affinity to FcγRs, avidity effects may also influence antibody effector functions. A recent study by Kao et al. showed that the decreased FcγR affinity that may occur due to changes in glycan structure, may be overcome by avidity. It was shown that IgGs with a mono- or disaccharide sugar domain, were sufficient to maintain cytotoxic antibody activity, suggesting that the binding of an antibody to its cognate antigen or avidity effects can overcome the loss in affinity that is observed by decreasing the glycan structure (22). However, the IgG glycovariants that we produced in our study do not reduce the level of IgG glycosylation beyond the mannose-rich central core. These glycovariants therefore do not abrogate IgG activity and still bind FcγRs (14).

The discrepancy between the results obtained using glycoengineered anti-RhD IgG to stimulate phagocytosis of erythrocytes, and those using the same glycovariants as irrelevant monomeric anti-TNP IgG antibodies to block opsono-phagocytosis cannot readily be explained. One remarkable conclusion is that defucosylation *per se* may not be essential. Only if in addition to defucosylation there is increased bisection or decreased galactosylation, the antibodies have increased inhibitory efficacy (Figure 2).

In this study we show for the first time that glyco-engineering can improve the blocking capacity of IgG, which is a promising tool to improve the immunomodulating efficacy of IVIg, when macrophages are being targeted. In case NK cells would be the cellular target to maximize IgG-mediated treatment efficacy, mere defucosylation may suffice (13). However, when macrophages are being targeted, increased bisection or decreased galactosylation is required in addition to defucosylation. Being a natural non-immunogenic component of normal plasma IgG, modified IVIg glycoproducts could potentially lead to a decrease in the therapeutic dose required or the number of IVIg infusions needed, which may also decrease the risk of side-effects, such as flu-like symptoms, headache, nausea, fatigue or IVIg-associated hemolysis (2, 3). It has to be shown whether the production of polyclonal IVIg preparations being either selected or engineered to contain a majority of selected IgG glycovariants will be technically feasible at large scale.

Moreover, our *in vitro* phagocytosis assay is a clean set up to study the interaction between FcγRs and IgGs. It is performed in the absence of human plasma, which contains high concentrations of IgG. Further *in vivo* studies should show if the glycovariants that were found to have an improved blocking potential *in vitro*, have a beneficial effect *in vivo* as well.

## Author contributions

CB designed and performed experiments, analyzed and discussed data and wrote the manuscript. GD designed and performed experiments, analyzed data. RV designed and performed experiments. NG performed experiments and analyzed data. TvdB, TR, GV, and TK designed the study and experiments, discussed data and wrote the manuscript. All authors contributed to reviewing the data and writing the manuscript.

### Conflict of interest statement

The authors declare that the research was conducted in the absence of any commercial or financial relationships that could be construed as a potential conflict of interest.

## Supplementary material

Supplementary Figure 1 Phagocytosis of opsonized erythrocytes is inhibited by anti-TNP IgG1 antibodies with different glycoforms in a dose-dependent manner in both M-CSF and GM-CSF cultured macrophages. Anti-TNP antibodies (in 0.1, 0.3, 1, 3, and 10 μg/mL, indicated on the x-axis) were used to inhibit the phagocytosis of anti-RhD opsonized erythrocytes by monocyte-derived macrophages cultured with GM-CSF **(left)** or M-CSF **(right)**. Data were normalized to the percentage of unblocked macrophages that phagocytosed anti-RhD opsonized erythrocytes. Data represent mean and standard error of the mean of one batch, representative for the other batches tested.

Supplementary Figure 2 Dimer content of anti-TNP IgG1. **(A)** Representative HP-SEC chromatograms of anti-TNP IgG1 run on HPLC Superdex 200 10/300 gel filtration column. Besides the large monomeric peak on the right, a small dimeric peak can be seen. **(B)** Dimer content of the different anti-TNP glycovariants (x-axis) does not correlate with the blocking capacity (y-axis), neither for the M-CSF- (circles) nor for the GM-CSF- (squares) cultured macrophages. Unmodified anti-TNP is shown in blue, anti-TNP –F+B (best blocking glycoform) is shown in red.

Supplementary Figure 3 Glycopeptide abundance of the –F+B and –F−G glycoforms. The glycosylation traits of the –F, –F+B, and –F−G glycoforms were calculated from the relative abundances of individual *N*-glycans (14). G, number of galactoses; F, presence of core fucose; N, presence of a bisecting *N*-acetylglucosamine; S, number of *N*-acetylneuraminic (sialic) acids. The presented data are from one technical replication. The glycoengineered antibodies were produced using the techniques we previously described (14, 18) that resulted in highly reproducible glycoprofiles for each glycoengineering method used.
