# Supplementary figures and images for: IgG Glyco-Engineering to Improve IVIg Potency
Source: Front Immunol. 2018 Oct 23;9:2442. doi: 10.3389/fimmu.2018.02442 (PMC6206079; doi:10.3389/fimmu.2018.02442)

Supplementary figure 1

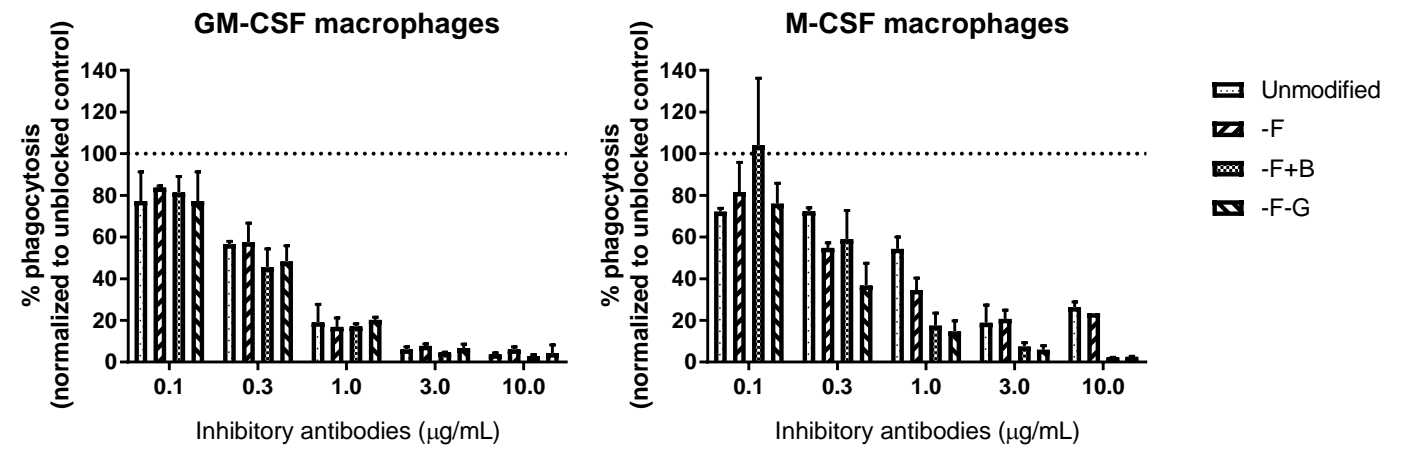

Supplement: Supplementary Figure 1 — Phagocytosis of opsonized erythrocytes is inhibited by anti-TNP IgG1 antibodies with different glycoforms in a dose-dependent manner in both M-CSF and GM-CSF cultured macrophages. Anti-TNP antibodies (in 0.1, 0.3, 1, 3, and 10 μg/mL, indicated on the x-axis) were used to inhibit the phagocytosis of anti-RhD opsonized erythrocytes by monocyte-derived macrophages cultured with GM-CSF (left) or M-CSF (right). Data were normalized to the percentage of unblocked macrophages that phagocytosed anti-RhD opsonized erythrocytes. Data represent mean and standard error of the mean of one batch, representative for the other batches tested. [file Image_1.PDF]

Supplementary figure 2

A

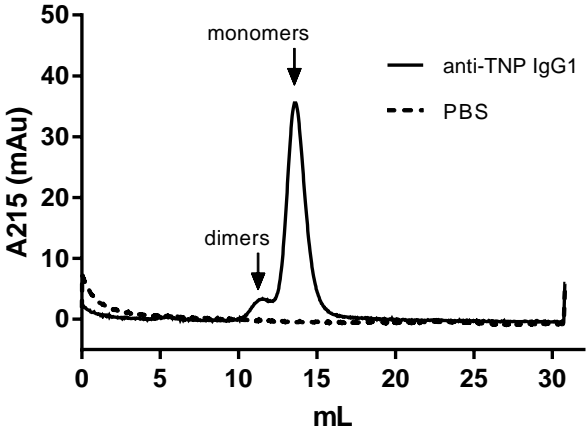

B

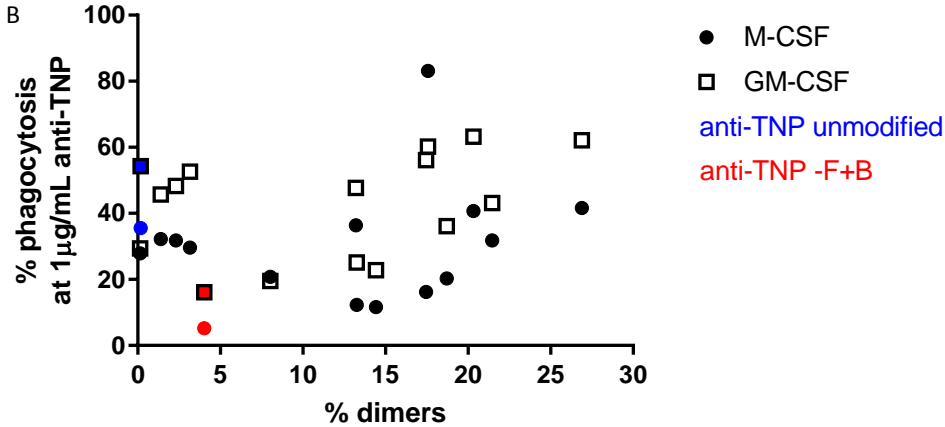

Supplement: Supplementary Figure 2 — Dimer content of anti-TNP IgG1. (A) Representative HP-SEC chromatograms of anti-TNP IgG1 run on HPLC Superdex 200 10/300 gel filtration column. Besides the large monomeric peak on the right, a small dimeric peak can be seen. (B) Dimer content of the different anti-TNP glycovariants (x-axis) does not correlate with the blocking capacity (y-axis), neither for the M-CSF- (circles) nor for the GM-CSF- (squares) cultured macrophages. Unmodified anti-TNP is shown in blue, anti-TNP –F+B (best blocking glycoform) is shown in red. [file Image_2.PDF]

Supplementary figure 3

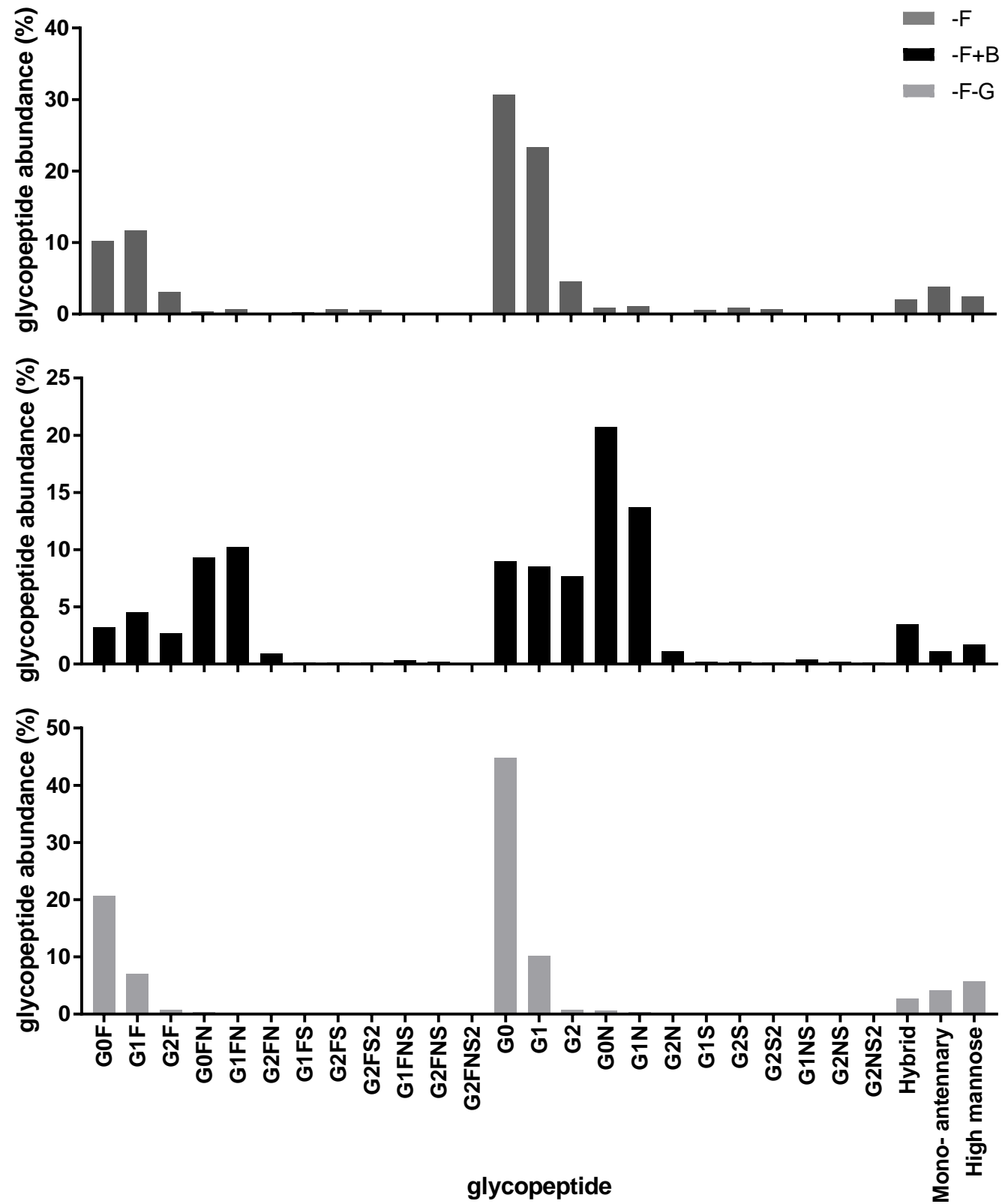

Supplement: Supplementary Figure 3 — Glycopeptide abundance of the –F+B and –F−G glycoforms. The glycosylation traits of the –F, –F+B, and –F−G glycoforms were calculated from the relative abundances of individual N-glycans (14). G, number of galactoses; F, presence of core fucose; N, presence of a bisecting N-acetylglucosamine; S, number of N-acetylneuraminic (sialic) acids. The presented data are from one technical replication. The glycoengineered antibodies were produced using the techniques we previously described (14, 18) that resulted in highly reproducible glycoprofiles for each glycoengineering method used. [file Image_3.PDF]
